# Voices from the Frontline: Understanding the Barriers and Enablers to Vaccination in Aged Care Facilities in Sydney, Australia

**DOI:** 10.3390/vaccines13111137

**Published:** 2025-11-04

**Authors:** Courtney McGregor, Lisa Maude, Karen Chee, Lauren Tillman, Caitlin Swift, Mark Ferson, Brendan Goodger, Kira Wright, Vicky Sheppeard

**Affiliations:** 1 South Eastern Sydney Public Health Unit, Sydney, NSW 2031, Australia; lisa.maude@health.nsw.gov.au (L.M.); karen.chee@health.nsw.gov.au (K.C.); lauren.tillman@health.nsw.gov.au (L.T.); caitlin.swift@health.nsw.gov.au (C.S.); mark.ferson@health.nsw.gov.au (M.F.); vicky.sheppeard@health.nsw.gov.au (V.S.); 2 School of Population Health, University of New South Wales, Sydney, NSW 2033, Australia; 3 Central and Eastern Sydney Primary Health Network, Sydney, NSW 2020, Australia; b.goodger@cesphn.com.au (B.G.); k.wright@cesphn.com.au (K.W.); 4 School of Public Health, University of Sydney, Sydney, NSW 2050, Australia

**Keywords:** vaccination in aged care, residential aged care facility, barriers, enablers, vaccine hesitancy, health policy, advocacy, qualitative methods

## Abstract

Background/Objectives: Vaccination is a critical public health measure for older adults in residential aged care facilities (RACFs). In Australia, COVID-19, influenza, pneumococcal, and shingles vaccines are recommended and funded for this group. However, vaccination coverage remains suboptimal, with limited understanding of the underlying causes. Methods: A mixed-methods design explored the enablers and barriers to vaccination from the perspectives of frontline providers, RACF staff, residents and family members. Descriptive statistics were used to quantify the prevalence of perceived enablers and barriers within stakeholder groups. Qualitative data—collected through open-ended questions—were analysed using manual, deductive-iterative coding to identify key themes. Key quotes illustrate the findings. Results: Input was gathered from seven in-reach geriatric staff, 40 general practitioners (GPs), 90 RACF staff, 17 RACF residents, and 84 family members of residents. Results were grouped under four key themes: operational, communication, coordination, and financial. RACF staff identified limited access to vaccination histories as the most significant barrier and relied on external providers to upload data to the Australian Immunisation Register (AIR). On-site clinics were essential, but organisational policies prevented nurse-led vaccination of residents. Most RACFs stored only influenza vaccines and depended on external providers for others. Simplified, translated information was called for. Healthcare provider and RACF endorsement was valued, but RACF staff felt ill-equipped to handle conversations around vaccine hesitancy. Consent processes were burdensome, and responsibility for tracking vaccination schedules was unclear with calls for streamlined processes. Low provider remuneration was also noted, with calls for increased government support. Conclusions: This work identifies key enablers and barriers to resident vaccination in RACFs. Improving delivery requires organisational policy change, staff support, digital access, and continued advocacy. Analysis of targeted interventions and coverage will be reported separately. The approach is replicable for other vulnerable groups.

## 1. Introduction

Immunisation is one of the most significant public health interventions globally, preventing illness, disability, and death from vaccine-preventable diseases (VPDs). Australia delivers one of the world’s most extensive and well-structured immunisation programs [1] through publicly funded vaccines that protect individuals across the lifespan, with a strong focus on vulnerable populations including older adults [2].

Older Australians represent a growing demographic, with 17.1% of the Australian population aged 65 years or older in 2022, an increase from 15.9% in 2018 [3]. Approximately 4.1% of older Australians permanently reside in residential aged care facilities (RACFs) [3], accounting for almost 190,000 Australians in 2024 [4], with an average age at admission of 85 years [5].

In Australia, COVID-19, influenza, pneumococcal and shingles (herpes zoster) vaccines are recommended and funded for older adults under the National COVID-19 Vaccine Program [6] and the National Immunisation Program (NIP) [7]. When these vaccines are administered, they are required to be uploaded to the Australian Immunisation Register (AIR) [8]. RACFs are encouraged to regularly review residents’ vaccination status, accessible via the AIR if the organisation employs an authorised nurse immuniser (ANI) (a registered nurse with additional vaccination training) or through My Health Record (MHR), provided the resident has not opted out [9]. At the time of this work, AIR access in RACFs was limited to facilities employing an ANI. Ensuring RACF residents are up to date with recommended vaccinations has dual benefits of protecting the individual from serious illness or hospitalisation and reducing the risk of infectious disease outbreaks within RACFs [10].

Current funded vaccination recommendations relevant to this population are listed in Table 1, however the proportion of older adults who receive these vaccines on time is below the New South Wales (NSW) Immunisation Strategy 2024–2028 targets [11].

As of April 2025, 20.6% of people aged ≥75 years and 10.3% of people aged 65–74 years in Australia had received a COVID-19 vaccine within the preceding six months [13]. Influenza vaccine coverage among older adults declined from 70.0% in 2022 to 61.7% in 2024. Although pneumococcal vaccination rates have shown a gradual year-on-year increase, coverage remains suboptimal; only 41.5% of adults aged 70 years and older were vaccinated as of 2024 [14]. Similarly, in 2023, just 41% of Australians turning 71 years had received at least one dose of the shingles vaccine [15].

Vaccination coverage data specific to residents of RACFs is limited, however does indicate substantially different patterns compared to the general population. As of April 2025, 44.4% of RACF residents in Australia had received a COVID-19 dose within the preceding six months [13]. In 2022, median RACF resident vaccination coverage in Victoria was 32.8% for pneumococcal vaccination and only 19.3% for shingles vaccination [16]. At that time in Australia a single dose of Zostavax shingles vaccine was funded on the NIP at ≥70 years, replaced by Shingrix in November 2023.

Vaccination coverage is shaped by a complex interplay of demographic, structural, social, and behavioural factors [17]. Low coverage in RACF residents specifically, has been linked to assumptions that residents are vaccinated before admission and the absence of mandatory government reporting requirements [8,16]. A systematic review of aged care staff practices identified barriers to residents’ general health outcomes, including high staff turnover and workload, limited staff education, lack of support from senior staff, infrastructure and logistical challenges, and resident and family attitudes. The review recommended considering barriers from staff, organisational, resident, family, and external perspectives to improve resident outcomes [18]—an approach adopted in this study, specifically targeting vaccination in South Eastern Sydney Local Health District (SESLHD) RACFs.

SESLHD comprises 97 RACFs that accommodate nearly 8000 residents. The district serves an overall population of almost one million people [19], with 40% born overseas, and 30% born in a non-English speaking country [20], highlighting the need for cultural responsiveness. In partnership with the Central and Eastern Sydney Primary Health Network (CESPHN), the SESLHD Public Health Unit (PHU) led a quality improvement initiative with the overall aim of improving the age-recommended vaccination coverage of COVID-19, influenza, pneumococcal and shingles vaccines for SESLHD RACF residents.

This paper presents the perspectives of frontline providers, RACF staff, residents and family members regarding enablers and barriers to vaccination of residents in SESLHD RACFs. By capturing insights from both implementers and end-users, this work contributes to the evidence base on improving vaccination uptake in aged care settings and informs future policy and practice.

## 2. Materials and Methods

### 2.1. Study Design and Data Collection

A mixed-methods design was used to explore vaccination practices, barriers, and enablers in RACFs. This approach combined quantitative and qualitative data collection through tailored questionnaires, allowing for both measurable trends and deeper insights into stakeholder experiences. The mixed-methods design was chosen to provide a comprehensive understanding of the issue from multiple perspectives, supporting the development of targeted interventions and resources.

Themes were based on the 5As framework (awareness, acceptance, access, affordability, and activation) [17] and refined based on material presented by the National Centre for Immunisation Research and Surveillance (NCIRS) [21]. Topics included consent, access to vaccination history and the AIR, access to providers, vaccination knowledge, and services funded or eligible under Australia’s national health insurance scheme (Medicare) [21]. Questions were adapted to ensure language, framing and context suited each stakeholder group, while maintaining consistent themes (Questionnaires S1).

Questionnaires were piloted internally by PHU staff not involved in their design.

Participants either self-completed their respective questionnaire online via REDCap or their verbal responses were transcribed into REDCap by a project team member in person or via telephone. Questionnaires were completed once, with no participant review of transcripts or findings. Data was collected between June and September 2024.

### 2.2. Participants and Recruitment

Five key stakeholder groups participated in the study. Informed consent was obtained from all participants prior to commencing the questionnaire. Participation was voluntary, responses were anonymous, and participants were informed they could skip questions if they wished. Data were stored securely on NSW Health-approved platforms, including REDCap. A convenience sample from each stakeholder group was invited using the following recruitment methods:

1. In-reach geriatric teams: The SESLHD Aged Care Stream manager emailed geriatricians and nurses who visit RACFs, inviting them to complete an online questionnaire within two weeks.
2. Local GPs: CESPHN emailed an online questionnaire link to their mailing list of approximately 340 GPs and promoted it in their newsletter, with a three-week response period.
3. RACF staff: facility contact lists held by the SESLHD Aged Care Stream manager and by the PHU were reconciled. Each of the 97 listed RACFs were contacted to confirm a key contact and invite participation. Questionnaires were either completed with the project team on-site or via telephone, or self-completed online. On-site visits were prioritised for facilities with low vaccination rates or a high proportion of residents born overseas. Verbal responses were transcribed by project staff to support RACF staff in completing the questionnaire, which was lengthy and potentially burdensome.
4. RACF residents: facility staff were asked to select cognitively competent residents able to provide informed verbal consent based on their knowledge of residents’ clinical and functional status, aiming to include vaccine-hesitant individuals where possible. No proxy consent was used. Ethical safeguards included one-on-one questionnaire completion conducted on-site by trained project staff, with sensitivity to residents’ comfort and autonomy. Verbal responses were transcribed by project staff to improve accessibility and inclusivity, particularly for residents who may have experienced literacy, vision, or physical limitations.
5. Family members of RACF residents: whilst we had hoped to complete questionnaires with family members during facility site visits, this was generally not feasible. RACFs were asked to email family contact lists a link to an online questionnaire, with responses collected over six weeks.

### 2.3. Data Analysis

Quantitative data was derived from closed-ended questions contained within all stakeholder questionnaires. Descriptive statistics were used to analyse the prevalence of perceived barriers and enablers within stakeholder groups. The percentages presented in the Results reflect quantitative survey responses.

Qualitative data—which were collected through open-ended questions—were analysed in a deductive and iterative manner [22]. Manual analysis was conducted using Microsoft Excel. Responses were organised by stakeholder group and initially broken down by pre-determined themes and topics described in Section 2.1.

Coding was conducted independently by two project team members. Discrepancies were discussed and resolved collaboratively to ensure consistency. While formal codebook and intercoder reliability metrics were not developed, the structured use of Microsoft Excel to document coding decisions and organise responses supported transparency and reproducibility.

As this was a quality improvement initiative, data saturation was not a goal; instead, the aim was to capture a wide range of stakeholder perspectives. Selected participant quotes were anonymised and included to illustrate key findings.

## 3. Results

### 3.1. Participants

Questionnaires were completed by seven staff from in-reach geriatric teams (three geriatricians, two nurse practitioners and two nurses) and 40 GPs. Staff from 90 of 97 (93%) RACFs completed questionnaires, with 53% completed during a site visit from the project team. These staff had various roles including facility managers, care managers, infection prevention and control leads, corporate staff and registered nurses.

Seventeen residents from 11 different RACFs completed questionnaires during site visits, and 84 family members of residents from 15 RACFs completed the family questionnaire online. Demographic details of residents or family members who completed questionnaires are in Table 2.

Of the 84 family respondents, 61 (74%) were adult children, 10 (12%) spouses and 11 (14%) another relation. For decisions regarding vaccination of the resident, 55 (66%) of the family respondents were the sole decision maker, 22 (27%) made joint decisions with the resident and three (4%) reported that the resident made vaccination decisions independently.

Barriers and enablers were categorised into four key themes: operational, communication, coordination, and financial. Table 3 outlines these themes and sub-themes, along with the consolidated recommendations from stakeholder groups.

### 3.2. Operational Barriers and Enablers

#### 3.2.1. Access to Residents’ Vaccination Histories

RACF staff ranked limited access to vaccination histories as the most significant barrier to vaccination of RACF residents. While 25 (28%) of the 90 participating RACFs employed an ANI, only 12 (13%) accessed the AIR. The remaining 13 facilities were unaware that employing an ANI allowed AIR access. MHR was available at 10 (11%) RACFs, but only 2 (2%) knew how to retrieve vaccination history using it.

“*unable to access AIR.… [RACF] cannot rely on GP to chase their old vaccination history. Some residents had multiple GPs prior to admission*.”

RACF 19

All RACFs relied on external vaccination providers to upload vaccination encounters to AIR. However, accurate and timely reporting to AIR was identified as an issue.

“*Often see that vaccinations are not correctly sent to AIR by pharmacy so have to chase them up*”

Family member 69

“*Outside agencies sometimes forget to put vaccines on AIR so difficult to know who has/has not had them*.”

GP 30

RACFs reported a strong need for direct access to residents’ immunisation histories, specifically using AIR. In-reach geriatric teams, GPs, and aged care staff agreed that this access would significantly improve vaccination tracking and delivery.

“*Difficult when new residents arrive at the facility, and they can’t provide documentation. This could be overcome with access to AIR*.”

RACF 17

#### 3.2.2. Access to Vaccination Providers and Organisational Vaccination Policies

On-site vaccination clinics were considered essential by RACF staff, residents and families due to mobility or frailty issues, with 85 (94%) RACFs reporting vaccinations were given mainly on-site.

“*Having the clinics run in the facility is the most helpful thing to access the vaccinations and prompt when the next vaccine is due*.”

Resident 16

Models of on-site vaccine administration varied considerably between facilities. RACFs used a combination of pharmacist immunisers (54/90, 60%), facility GPs (46/90, 51%), residents’ own GPs (32/90, 36%) and ANIs (13/90, 14%). Nine (10%) RACFs reported difficulty securing a vaccination provider to attend on-site.

Having access to multiple vaccination providers was widely considered a key enabler of vaccination for RACF residents. Many respondents highlighted the benefits of on-site providers who were familiar with residents, especially those with cognitive dysfunction.

“*Best done within the facility by people experienced with dementia*.”

Family member 83

Many RACFs were shifting to using external pharmacist immunisers for on-site vaccination clinics, citing inefficiencies with multiple GPs each vaccinating only their own patients and the administrative burden of uploading vaccine records to the AIR. However, some GPs were uncomfortable with this approach.

“*Pharmacists are a poor substitute to a registered nurse who has clinical bedside training. Our RACF system has disempowered nurses and overall the system is now crumbling*”

GP 32

Although 25 (28%) RACFs employed an ANI, only 13 of the 25 (52%) facilities utilised them for resident vaccinations, due to RACF policies preventing them from administering vaccines to residents and lack of access to AIR.

“*ANI gives staff vaccines but not confident to give resident vaccines as they have no way to enter data on AIR*.”

RACF 34

“*She is qualified [to vaccinate residents] but the RACF does not have the correct policy to allow for this*.”

RACF 70

Nurse practitioners were employed at 30 (33%) RACFs. GPs were generally supportive of nursing staff expanding their role to include resident vaccination and some expressed frustration that nurses were not vaccinating, possibly unaware that this was due to policy restrictions rather than individual choice.

“*Every RACF has a registered nurse—they should be promoted to vaccinate residents*.”

GP 32

“*The biggest barrier in my opinion is the resistance of the nurse practitioners and nurses within the nursing facility who don’t want to administer vaccines. However, they are trained clinical staff who are giving them medications anyway, so I don’t understand why the nurse practitioners or nurses in care homes are reluctant to administer vaccines*.”

GP 4

Twenty (22%) RACFs (generally smaller size facilities) did not have a formal vaccination policy for residents, and several of these requested examples of key components to include in such documents.

“…*[we] would appreciate a sample vaccination policy*….*[we] do not have the vaccination policy*”

RACF 18

#### 3.2.3. Access to Vaccines

Of the 90 participating RACFs, 80 (89%) had a vaccine fridge, but most only ordered and stored influenza vaccines, and relied on providers to bring other vaccines on-site. RACFs could not order COVID-19 vaccines due to Australian government policies. GPs expressed frustration about being expected to supply vaccines themselves.

“*…Another RACF supplies the flu vaccine and lets me do it, but then they did not stock COVID vaccine and were expecting me to bring [it] from practice…*”

GP 30

“*The major barrier to increasing vaccination rates for me is access to vaccines, in particular to facilities without a cold chain. In these instances, I am expected to provide vaccines… In facilities in which there is a cold chain and the facility manager is able to order the correct amount of flu/pneumo/zoster [shingles], my patients are 100% up to date*”

GP 13

Removing the State Vaccine Centre ordering limits for vaccination providers running clinics for RACFs was seen as important, with suggestions for:

“*an exemption or an alternate system for providers who visit RACFs to enable increase volume of particular vaccines to be ordered [is required]. This is such a simple intervention*.”

GP 13

Of the 40 GP respondents, 34 (87%) reported they would be more likely to administer vaccines to RACF residents if the vaccines were readily available on-site, making it logistically easier to run clinics:

“*RACF should have vaccines on site in proper fridges*”

GP 25

“*Have vaccines stored at the RACFs with appropriate needles, cotton balls, small sharp containers and bandaids available*.”

GP 19

### 3.3. Communication Barriers and Enablers

#### 3.3.1. Vaccination Information

RACF staff reported difficulty accessing translated vaccination resources, particularly relating to the shingles and pneumococcal vaccines. This limited their ability to support informed decision-making among residents from culturally and linguistically diverse (CALD) backgrounds.

Of the participating residents, 5 of 14 (36%) felt they had not received enough information about vaccines. Among 84 family respondents, 56 (67%) relied on healthcare providers, 40 (48%) on RACF staff, and 36 (44%) on media sources (e.g., TV, radio) for vaccine information.

Both residents and families expressed a desire for clearer, more specific and accessible information.

“*Genuinely HONEST information and SPECIFIC data… The current sheet just says—it is recommended and very safe*.”

Family member 80

“*Media plays an important role… speak in layman terms so people can understand*.”

Resident 8

Family influence was a key barrier to vaccine uptake, particularly when concerns were raised about the recommended six-month interval for COVID-19 vaccination or when there was mistrust in vaccines.

“*Primarily hesitancy or refusal is coming from the family*”

RACF 12

“*COVID and flu vaccines do not work… More thorough research into the truth…*”

Family member 78

Resident vaccination hesitancy was linked to fear of side effects, and behavioural issues, particularly among those with dementia.

“*Even though there is consent, the immuniser cannot proceed, as they are unable to restrain [the resident]*.”

RACF 12

#### 3.3.2. Patient-Provider Consultation

Of the GP respondents, 37 of 39 (95%) provided on-site consultations and 32 of 37 (86%) administered vaccinations to residents in RACFs. However limited time for vaccine education was noted by in-reach geriatric teams and GPs, with visits largely occurring when residents were acutely unwell.

“*GPs try hard to attend to give vaccinations… but have little time to provide education… to residents or their family/person responsible*.”

In-reach geriatric team member 4

“*Focus of care is more on treatment of current episode rather than prevention*.”

In-reach geriatric team member 3

“*Patient care in RACF is reactive… not focused on preventative health*.”

GP 9

This meant that RACF staff were often relied upon to initiate discussions with residents and families, even though they felt ill-equipped and attempted to defer vaccine hesitancy conversations to medical providers.

“*The staff have been trying to address misinformation/vaccine hesitancy by circulating resources to help with informed decision making. They seem burnt out by continual obstruction from families though, so reported rarely having individualised discussions*.”

RACF 30

“*Encourage GP to have conversation if resident is vaccine hesitant*.”

RACF 6

Residents highlighted the importance of trust in RACF staff, and the value of education and endorsement was widely acknowledged, especially when families were hesitant or unsupportive of vaccination.

“*RACF staff play a big role*”

Resident 7

“*Education to carers and family members on the importance of vaccination [is required], and answering any concerns to minimise hesitation or refusal*”

In-reach geriatric team member 3

“*Relatives need more education so they don’t refuse vaccines on behalf of residents*.”

Resident 13

Many family members echoed the importance of healthcare provider endorsements in the decision-making process:

“*My father’s GP always recommends what vaccinations are due*.”

Family member 25

“*All has to be consulted with the health staff and GP, then weigh up all the reasons and benefits of these vaccinations, [acceptance of vaccination] will be decided accordingly*.”

Family member 44

#### 3.3.3. Consent

Organising vaccination consent was reported as a major barrier by all respondent groups, particularly for residents unable to provide consent themselves.

“*It is difficult at times to get consent. Where the patient is unable to, it can be difficult to get on to next-of-kin and they don’t answer emails*”

GP 36.

It was noted to be particularly time-consuming for residents under Public Guardianship.

“*Issues obtaining consent from Public Guardian*—*[they] never reply regarding consent*”

RACF 1

Consent delays were common, sometimes due to the Public Guardian requesting GPs or geriatricians complete a “consent for medical treatment” form. Additional delays arose from separate forms for each vaccine, unresponsive families, and GPs requiring renewed consent despite earlier collection. Verbal phone consent was a common but time-consuming workaround.

“*Often have to wait for significant number of residents to consent for the vaccination process to be carried out. Most recently it was a two-month wait from date of sign off until vaccination*”

Family member 35

“*Will send emails to families or seek verbal consent and document accordingly. Have found verbal consent to work better, as families tend to forget to send back the consent form*”

RACF 11

Consent for pneumococcal and shingles vaccination was not routinely obtained by RACFs and largely viewed as the GP’s responsibility. Streamlined consent processes were called for, with a common proposal being a single, modifiable form covering multiple vaccines.

“*Help facility develop generic consent form for all 4 vaccinations*.”

RACF 1

“*One consent form to cover regular COVID-19 and influenza vaccines, rather than having to ask each time*.”

RACF 28

The use of digital tools to simplify consent was also recommended, including web-based forms and electronic submission options.

“*A web form that you can complete and submit online is essential*.”

Family Member 45

“*Identify ways to improve problems around accessing consent from families and the guardianship board… electronic options*.”

RACF 87

### 3.4. Coordination Barriers and Enablers

#### Tracking Vaccination Schedules

All respondent groups recognised the value of RACFs actively tracking vaccination schedules and informing GPs and family members when vaccines were due.

“*The RACF could monitor the vaccination status and notify GPs when vaccines are due… as is already happening in some RACF facilities that I visit*”

GP 29

“*Having the facility let us know when vaccinations are due is really helpful, and they organise for the nurse/doctor to administer it at the facility*”

Family member 68

Most RACFs tracked residents’ COVID-19 (98%) and influenza (99%) vaccinations manually, but only 46% monitored pneumococcal and shingles uptake. Tracking was often left for GPs, leading to role confusion noted by families.

“*Poor in-house systems meant one vaccine was missed when due & requested*”

Family member 8

“*Lack of clarity regarding who is responsible for organising the vaccination—i.e., is the family or the facility going to arrange this*?”

Family member 72.

Residents reported reliance on facility staff to keep them informed about when they are due for vaccinations and healthcare providers highlighted the importance of individualised reminders tailored to each resident’s vaccination schedule, as opposed to generic notifications.

“*Having reminders for RACF or the GP for any due age-recommended vaccinations for the resident is helpful. This is better than a generic reminder for all*”

In-reach geriatric team member 3

GPs also expressed concerns about the fragmented tracking and communication, noting:

“*No tracking in RACF software. Difficult to determine if they have been done or not. Also, they get outside agencies to do COVID and flu but not shingles and pneumococcal vaccines. No communication between facilities and GPs*”

GP 7

“*I’d rather the aged care homes track it, and wish they could access the immunisation register*”

GP 3

Maintaining an up-to-date vaccination register was seen as vital, but RACFs lacked a unified system to track all four vaccines. Separate reports, manual calculations, and multiple software platforms were being used.

“*COVID and flu is checked manually for each resident when arranging an upcoming clinic, no automated system, and report these numbers to the Quality team [in Head Office]*.”

RACF 47

### 3.5. Financial Barriers and Enablers

#### Government Reimbursement

Multiple aged care staff highlighted a lack of GPs willing to come on-site to administer vaccinations. Of the GP respondents who did not administer on-site vaccinations, two (33%) cited the lack of payment and confusion over government reimbursement for vaccinating residents in RACFs as one of the reasons.

“*… It is unclear what item numbers can be used for a facility visit to give COVID [vaccination] as well as others*”

GP 36

Financial incentives were seen by both in-reach geriatric teams and GPs as a key enabler to improve GP engagement in aged care vaccination, particularly given workforce shortages and increasing resident numbers.

“*Making the [vaccination] discussion with family a billable encounter could be an incentive for the GP*.”

In-reach geriatric team member 3

“*Agree with increased GP incentives. Dwindling number of GPs with more patients in nursing homes*.”

GP 40

One GP suggested redirecting incentives to RACFs themselves to encourage vaccination uptake at the facility level:

“*Maybe incentives for the RACF to have their residents vaccinated*.”

GP 27

## 4. Discussion

This study highlights key operational, communication, coordination, and financial factors influencing vaccination uptake in RACF residents. Drawing on perspectives from frontline providers, staff, residents, and families, it offers recommendations to inform policy development, practice improvements, and advocacy in aged care settings.

### 4.1. Operational Considerations

Lack of access to residents’ vaccination histories was a key issue, as this information was not readily available from GPs or family members upon admission to a RACF. Therefore, enabling RACF staff to access either AIR or MHR is essential. The issue of underused health information databases and systems in RACFs has been reported before [23]. Our study found that ANIs working in RACFs were not aware they could access AIR to view residents’ immunisation histories. The MHR system, an alternative database for RACFs that did not employ ANIs, was also under-utilised due to lack of staff awareness. These results show that RACF staff require clear instructions, ongoing education and support on how to access such systems to enable monitoring of RACF resident vaccination status. In response to these findings, the project team successfully advocated for a nationwide policy change, resulting in all RACFs having the ability to apply for direct access to the AIR regardless of ANI employment, as of May 2025 [24].

On-site vaccination using a range of vaccination providers was considered important. GPs and in-reach geriatric teams endorsed nurse-led vaccination in RACFs; however, organisational policies often prevented ANIs from vaccinating residents, despite being appropriately qualified to do so. While the reasons for this were not explored in detail, similar organisational policy barriers have been noted previously [25]. Nurse practitioners were also found to be underutilised, consistent with existing literature [26]. Shifting organisational policies towards nurse-led vaccination would require RACF board-level endorsement, sustained support for frontline staff [27], and would likely benefit from encouragement from the Australian Department of Health, Disability and Aging.

When it comes to accessing vaccine stock, RACFs in Australia are not authorised to order or store COVID-19 vaccines independently and must rely on vaccination providers to do so via the national Vaccine Operations Centre [28]. While this issue was primarily raised by GPs in our study, the restriction likely presents logistical challenges for other vaccination providers as well—particularly when transporting large quantities of COVID-19 vaccines for mass clinics. In response, the project team has been advocating nationally to remove this restriction. At a state level, advocacy resulting from this project has led to increased NIP vaccine (influenza, pneumococcal and shingles) ordering limits for RACFs, small GP practices and pharmacies in NSW to support on-site mass vaccination clinics. However, access to appropriate cold chain management remains a key consideration. RACFs would benefit from enhanced education and guidance to ensure compliance with national cold chain standards [29].

### 4.2. Communication and Coordination Considerations

Our findings highlight the need for simplified and translated vaccination information, consistent with previous research in hospitals, long-term care, and among CALD and disadvantaged populations [30]. Barriers such as limited health promotion resources and insufficient time for patient-provider discussions have also been reported in rural and regional Australian settings [31]. To support informed decision-making, clear and accessible vaccine information is needed for RACF residents and families, alongside education for RACF staff to facilitate conversations about vaccination, particularly with vaccine hesitant individuals. Multi-component interventions with ongoing reinforcement, such as targeted information and education, have been shown to improve health outcomes for residents in RACFs, especially when linked to changes in staff behaviour [18]. On-site education has been identified as the most effective approach, increasing staff confidence in vaccination discussions [32]. Provider endorsement was also identified as a key influence on vaccination decisions for RACF residents and their families, aligning with other studies showing that recommendations from trusted health professionals increases vaccine uptake [30], particularly among CALD populations [33]. This is especially relevant in our District, where many RACFs serve residents from multiple ethnic backgrounds.

As the aged care sector faces increasing demand in Australia [3], there is a growing need for use of health information technologies that enhance care quality, efficiency, and workforce productivity. Electronic documentation systems, already adopted by most RACFs, improve the accuracy of records, access to information, and decision-making [23]. In our study, consent and tracking of vaccination schedules emerged as major barriers to resident vaccination, with consent challenges previously recognised in the literature [25,34]. New or existing systems incorporating online consent and tracking capabilities may help streamline administrative workflows, ensuring staff are informed, and support timely vaccination of RACF residents. However, barriers to implementing digital innovations in aged care include financial and time constraints [23,27]; resource demands [23,27,35]; technology infrastructure limitations [23]; and staff capability to use new systems [23,27,35]. Enablers include effective communication, adequate staff education, and organisational support through mentors or champions [23,27]. Collaborating with aged care organisations to develop and implement supportive policies is crucial for successful adoption [27].

### 4.3. Financial Considerations

Inadequate remuneration has been identified as a key barrier to GPs providing preventive care for more medically complex residents in RACFs [36]. Our findings support this, highlighting financial barriers including lack of reimbursement for vaccinations administered in RACFs, remuneration being directed to practices rather than individual GPs, and vaccine-related discussions not billable under current funding models. The Australian Government introduced the General Practice in Aged Care Incentive on 1 July 2024, aimed at supporting GPs and practices to deliver health care to permanent RACF residents [37]. Under this scheme, incentive payments are made to eligible GPs and practices quarterly, provided requirements for care planning services and regular visits are met [37]. This scheme was introduced around the time of our project data collection, meaning further work will be required to assess its effectiveness at increasing GP engagement in RACF resident vaccination.

### 4.4. Strengths and Limitations

A key strength of this work was the inclusion of diverse stakeholder perspectives, including frontline providers, RACF staff from a large proportion (93%) of facilities in the district, and residents and families from CALD backgrounds who are often under-represented in health research despite facing poorer health outcomes [20,38]. Among the participating residents, 41% were born overseas, aligning with the demographic profile of SESLHD [20], but notably higher than the national proportion in 2024, which was 31.5% [39]. Including these perspectives enhances the local relevance of our findings while contributing valuable insights applicable more broadly across Australia.

Despite these strengths, we acknowledge some limitations. Convenience sampling and recruitment via RACF managers may have introduced selection bias, and findings are not generalisable. Resident participation was low (only 17 participants), largely due to consent challenges and limited staff availability, with vaccine-hesitant residents underrepresented. Face-to-face questionnaire completion occurred in only 53% of RACFs, potentially excluding other eligible resident participants.

The GP survey had a low response rate (12%), resulting in possible non-response bias. Family member recruitment relied on RACF mailing lists, and the questionnaire—initially designed for face-to-face completion—may have been unclear when self-completed, leading to skipped questions and limiting linkage to specific RACFs.

Data collection via self-completed questionnaires or project team transcription of verbal responses into REDCap may have introduced response or transcription bias. Additionally, the use of open-ended questions instead of individual interviews may have reduced the depth of qualitative insights.

Finally, the Public Health Unit’s dual role in conducting the study and leading subsequent interventions may have introduced social desirability and interpretive bias.

### 4.5. Impact and Future Directions

In response to the insights gained from this work, the PHU developed a series of targeted interventions and supportive resources including an online consent template; vaccination action plan toolkit; a vaccination tracker register for residents; low literacy, translated factsheets [40]; a vaccination policy template; instructional videos to support intervention use [41]; and a series of online and face-to-face education sessions for RACF staff. These were delivered to participating RACFs between August 2024 and June 2025. Due to the breadth of data collected, we plan to present the targeted interventions and supportive resources, along with pre- and post-intervention vaccination coverage findings, in a separate publication.

Future work would benefit from direct engagement with RACF corporate bodies to address policy barriers, such as prohibiting nurse-led vaccination of residents and digital consent processes. Exploring enablers and barriers with pharmacist immunisers directly may also provide additional valuable insights into vaccination delivery in RACFs. Beyond RACFs, these methods are applicable to other vulnerable groups, including residents of independent living units (retirement villages) and disability group homes, who may face similar challenges. Strengthening engagement with broader stakeholder groups and vulnerable communities will ensure inclusive, tailored strategies to improve vaccine equity and help inform national immunisation strategies and policy.

## 5. Conclusions

This project has provided greater understanding of the complex barriers and enablers contributing to suboptimal vaccination coverage of RACF residents. Our findings show that strengthened and sustainable vaccination delivery in RACFs will require organisational policy changes; better support, education and training for RACF staff; improved RACF access to digital tools; and continued advocacy to government. Targeted interventions and vaccination coverage will be examined in a future manuscript. The methods used in this project offer a replicable framework for understanding vaccination coverage gaps in other vulnerable population groups.

## Figures and Tables

**Table 1:** Vaccines for older adults funded through the National COVID-19 Vaccine Program and the National Immunisation Program (NIP) [12].

Vaccine	Recommended Timing of COVID-19 Vaccines Booster Doses for Older Adults		
COVID-19	Every 6 months for all adults ≥ 75 years old Every 6 months for RACF residents ≥ 65–74 years old Every 12 months for general population ≥ 65–74 years old		
**Vaccine**	**Recommendation**	**Age Vaccine is Funded From**	
		**Non-Indigenous**	**Indigenous**
Influenza	Annual single dose	65 years	6 months
Pneumococcal Prevenar 13 (13vPCV) Pneumovax 23 (23vPPV)	Single dose of 13vPCV for all adults PLUS 2 doses of 23vPPV for all Indigenous adults and for non-Indigenous adults with vulnerable conditions	70 years	50 years
Shingrix	2 doses	65 years	50 years

**Table 2:** Demographic characteristics of RACF residents who completed the questionnaire in person (n =17) or whose family members completed the online questionnaire (proxy) (n = 84).

Resident Demographic Characteristics	N (%)	N (%)
	Resident completed questionnaire	Questionnaire completed by proxy
Sex		
Male	6 (37)	20 (22)
Female	11 (63)	64 (78)
Age		
65 to 69 years	0	1 (1)
70 to 74 years	3 (18)	5 (6)
75 to 79 years	3 (18)	11 (13)
80+ years	11 (64)	66 (80)
Indigenous status		
Aboriginal and/or Torres Strait Islander	1 (6)	0
Country of birth		
Australia	10 (59)	42 (50)
Overseas	7 (41)	42 (50)
Language spoken		
English	16 (94)	71 (80)
Other	5 (29)	21 (26)
Time lived in RACF		
<6 months	2 (12)	13 (16)
6 months to 1 year	2 (12)	14 (17)
1 to 3 years	8 (47)	34 (41)
>3 years	5 (29)	22 (26)

**Table 3:** Overview of themes and consolidated recommendations from stakeholders.

Theme	Sub-Theme	Recommendations
Organisational	Access to vaccination history	Direct access to AIR for all RACFs
	Access to vaccination providers & organisational policies	On-site vaccination clinics Nurse-led vaccination Vaccination policy template
	Access to vaccines	On-site vaccination storage Direct access to COVID-19 vaccines for RACFs Increase vaccine ordering capacity
Communication	Vaccination information	Low literacy, translated factsheets
	Patient-provider consultation	Healthcare provider endorsements Enhance RACF role in vaccine education
	Consent	Single, editable digital form covering multiple vaccines
Coordination	Tracking vaccination schedules	RACFs actively maintaining vaccination register
Financial	Government reimbursement	Financial incentives for GPs or RACFs

## Data Availability

The data presented in this study are available upon request from the corresponding author. Due to ethical restrictions, they are not publicly available.

## Supplementary Materials

The following supporting information can be downloaded at: https://www.mdpi.com/article/10.3390/vaccines13111137/s1, Questionnaire S1: All stakeholder group questionnaires.

## Abbreviations

The following abbreviations are used in this manuscript:

RACF	Residential aged care facility
NIP	National Immunisation Program
AIR	Australian Immunisation Register
ANI	Authorised nurse immuniser
MHR	My Health Record
NSW	New South Wales
13vPCV	Prevenar 13
23vPPV	Pneumovax 23
SESLHD	South Eastern Sydney Local Health District
CESPHN	Central and Eastern Sydney Primary Health Network
PHU	Public Health Unit
GP	General practitioner
NCIRS	National Centre for Immunisation Research and Surveillance
CALD	Culturally and linguistically diverse
