# Supplementary material for: Voices from the Frontline: Understanding the Barriers and Enablers to Vaccination in Aged Care Facilities in Sydney, Australia
Source: Vaccines (Basel). 2025 Nov 4;13(11):1137. doi: 10.3390/vaccines13111137 (PMC12656986; doi:10.3390/vaccines13111137)
Supplement: Supplementary file 1 [file vaccines-13-01137-s001.zip › vaccines-3907351-supplementary.pdf]

RACF resident vaccination project - SESLHD geriatricians & GFS questionnaire

Thank you for your opinions on the barriers and potential solutions to vaccinating residential aged care facility (RACF) residents. Your perspectives will help the Public Health Unit develop practical solutions to help RACF staff ensure their residents receive age-recommended vaccines in a timely way.

Your participation in this survey is voluntary, and your responses are confidential and anonymous.
If you do not wish to answer a question, please leave it blank.

If you have any questions about this survey or comments, please contact Karen Chee at the Public Health Unit (karen.chee@health.nsw.gov.au).

Thank you very much.

I consent to participating in this survey.
- ◯ Yes
- ◯ No

What is your role in SESLHD?
- ◯ Nurse
- ◯ Geriatrician
- ◯ Other

Please specify role: ______________________________

Do you routinely remind your patients who live in RACFs (or their family members) to obtain their age-recommended vaccinations (i.e. COVID-19, influenza, pneumococcal and shingles)?
- ◯ Yes, all age-recommended vaccinations
- ◯ Yes, some of the age-recommended vaccinations
- ◯ No

Why do you routinely remind your patients of some age-recommended vaccinations only?
- ◯ Not aware of all the age-recommended vaccinations
- ◯ Don't have time to discuss all the vaccinations
- ◯ Other

What is/are your other reason(s)? ______________________________

What prevents you from routinely reminding your patients about vaccinations? ______________________________

What do you think prevents RACF residents from receiving their vaccinations? (Please select all that apply)	☐ Lack of RACF staff awareness of need for vaccination<br>☐ Lack of resident awareness of need for vaccination<br>☐ Resident or family hesitancy or refusal<br>☐ RACF staff not aware which vaccinations a resident is due for<br>☐ RACF staff not able to access Australian Immunisation Register<br>☐ Challenges in obtaining consent<br>☐ Difficulty finding a vaccination provider (e.g. GP)<br>☐ Lack of vaccine fridge in RACF<br>☐ Lack of RACF staff knowledge in cold chain management<br>☐ Other
Can you please explain?	__________________________________
What do you think are the major barriers to vaccinating RACF residents that the Public Health Unit can work on?	__________________________________
Can you suggest ways to overcome some of the barriers?	__________________________________
Do you have any other comments on vaccinating RACF residents?	◯ Yes<br>◯ No
Can you please explain?	__________________________________

Vaccinating residential aged care facility residents - GP questionnaire

Thank you for your opinions on the barriers and potential solutions for ensuring residential aged care facility (RACF) residents receive their age-recommended vaccinations.

Your perspectives will help the Public Health Unit develop practical solutions to help RACF staff ensure their residents are vaccinated in a timely way.

Your participation in this survey is voluntary, and your responses are confidential and anonymous.
If you do not wish to answer a question, please leave it blank.

If you have any questions about this survey or comments, please contact Dr Karen Chee at the Public Health Unit (karen.chee@health.nsw.gov.au).

Thank you very much.

I consent to participating in the survey.	◯ Yes<br>◯ No
Do you provide in-home consults to patients who live in residential aged care facilities (RACF)?	◯ Yes<br>◯ No
Do you routinely remind your patients who live in RACFs (or their carers) to obtain their age-recommended vaccinations (e.g. COVID-19 every 6-12 months, influenza annually, herpes zoster at age 65, and pneumococcal at age 70)?	◯ Yes<br>◯ No
Do you have a system that tracks when patients are due for their vaccinations?	◯ Yes<br>◯ No
What is the name of the system or software you use?	__________________________________
Do you administer vaccinations to your RACF patients in their home?	◯ Yes<br>◯ No
What are the reasons for not administering vaccinations in RACFs?<br>(Please select all that apply)	☐ I do not provide in-home visits<br>☐ It is difficult for me to bring the vaccine(s) to the RACF<br>☐ The patient is usually too unwell to have a vaccination when I review them in their home<br>☐ No Medicare reimbursement for vaccinating RACF residents<br>☐ Other
What are the other reasons?	__________________________________

Where do you source the vaccinations for your RACF patients on the day of vaccination?	☐ From own practice<br>☐ From a pharmacy close to the RACF<br>☐ Available on-site at the RACF<br>☐ Other
Where else do you source your vaccines from?	____
If vaccines were available on-site in RACFs, would you be more likely to administer vaccinations to your patient(s)?	○ Yes<br>○ No<br>○ Don't know
Do you know that participating pharmacists can now administer vaccinations to RACF residents?	○ Yes<br>○ No
Would you recommend your patients have their vaccinations at pharmacies if they were unable to obtain them from your practice (e.g. no available appointments when vaccine is due)?	○ Yes<br>○ No
Can you explain why you would not recommend your patients have their vaccinations administered at pharmacies?	____
Do you have other suggestions on why it is difficult to keep RACF residents up to date with their recommended vaccinations?	____
Do you have any suggestions on how to ensure RACF residents receive their age-recommended vaccinations on time? (e.g. GP incentives from the Commonwealth)	____

RACF resident vaccination project - RACF staff questionnaire

PROVIDE THE INFORMED CONSENT LETTER.

The Public Health Unit is hoping to find out from people who work in aged care facilities what the issues and possible solutions are in making sure residents receive their recommended vaccinations on time.

Thank you for agreeing to take part in the project.

Your answers are confidential, and will not be shared with other staff or residents at your facility.

You do not have to answer any questions if you do not wish to.

You can stop at any time.

Do you have any questions before we start?

I consent to participating in this survey.
- ◯ Yes
- ◯ No

What is the name of the RACF? ______________________________

Name of RACF staff member interviewed (optional) ______________________________

Role of RACF staff member interviewed (select all that apply):
- ☐ Facility manager
- ☐ Registered nurse
- ☐ Assistant in nursing or carer
- ☐ Infection and prevention control practitioner
- ☐ Corporate level staff
- ☐ Other
- ☐ Care Manager

What is their role? ______________________________

Date of interview ______________________________

Name of PHU staff completing the interview ______________________________

Do you know that adults need 4 specific vaccinations when they turn a certain age?

• COVID-19 (due every 6 months for ≥75 yo; 6-12 months for 65-74 yo)
• Influenza (due annually at 65 yo for non-Indigenous adults or any age for Indigenous adults)
• Pneumococcal (due at 70 yo for non-Indigenous adults, or 50 yo for Indigenous adults)
• Shingles (due at 65 yo for non-Indigenous adults, or 50 yo for Indigneous adults)

	Yes	No
COVID-19	◯	◯
Influenza	◯	◯
Pneumococcal	◯	◯
Shingles	◯	◯

Further comments: ______________________

Do you have residents under the age of 65 living in your facility?
(If yes, provide the Factsheet for Vaccinations recommended for people under 65 with medical conditions)

◯ Yes
◯ No

How do staff feel about vaccinations for their residents?

	Very positive	Positive	Neutral	Negative	Very negative
Staff opinion on vaccinations for residents	◯	◯	◯	◯	◯

Further comments ______________________

Is there a staff member in the facility allocated to record resident consent for vaccinations?

	Yes	No	Don't know
Every 6 months for the COVID-19 vaccine	◯	◯	◯
Every year for the influenza vaccine	◯	◯	◯
When a resident turns a certain age for the shingles vaccine	◯	◯	◯
When a resident turns a certain age for the pneumococcal vaccine	◯	◯	◯

Further comments (optional): ______________________

How does your facility obtain consent for vaccinating residents?
(Select all that apply)

☐ Consent form developed by the facility
☐ Generic consent form
☐ Other
☐ Don't know

Comments for obtaining consent (if applicable): ______________________

What other method(s) does your facility use to obtain consent to vaccinate residents? ______________________

Do you use any Commonwealth or NSW Health vaccination resources for RACFs (e.g. consent flowchart)?
(Show examples of resources)

- ◯ Yes - Commonwealth resources
- ◯ Yes - NSW Health resources
- ◯ Yes - Commonwealth and NSW Health resources
- ◯ No
- ◯ Don't know

Which ones? ______________________

Further comments (optional) ______________________

Is the resident's vaccination history and consent included in their medical histories?

- ◯ Yes
- ◯ No
- ◯ Don't know

In what form is the vaccination consent?
(Select all that apply)

- ☐ Paper
- ☐ Electronic
- ☐ Other
- ☐ Don't know

Any other comments about vaccination history (if applicable): ______________________

What other form is the vaccination consent? ______________________

If a resident is not capable of giving consent, how do you obtain consent from their family or representative?
(Select all that apply)

- ☐ Over the phone
- ☐ By email
- ☐ Other

What other way do you obtain consent from a resident's family or representative? ______________________

How do staff manage the situation if residents or their families are unsure if they wish to have a vaccination or say they are fed up with having so many vaccines? ______________________

What other problems do you have when obtaining resident consent for vaccination? ______________________

Do you find consent issues cause big problems when vaccinating residents?	◯ Yes<br>◯ No<br>◯ Don't know
Further comments (optional):	__________
Are resident's vaccination status routinely recorded when they are admitted to the facility?	◯ Yes<br>◯ No<br>◯ Don't know
Any other comments about recording vaccination status (if applicable):	__________
Are your residents' Indigenous status recorded?	◯ Yes<br>◯ No<br>◯ Don't know
Does your facility have a vaccination policy?	◯ Yes<br>◯ No<br>◯ Don't know
Any comments about facility's vaccination policy (if applicable):	__________
Can you provide a copy of the policy?	__________
Does your facility have an infection prevention and control (IPC) lead?	◯ Yes<br>◯ No<br>◯ Don't know
Is your IPC lead involved in resident vaccination?	◯ Yes<br>◯ No<br>◯ Don't know
How is your IPC lead involved in resident vaccination?	__________
Does a pharmacist visit the facility for face to face medication reviews with either the resident or their family?	◯ Yes<br>◯ No
Do staff want direct access to residents' immunisation history?	◯ Yes<br>◯ No<br>◯ Don't know

Are you aware that staff can access residents' immunisation history using either the Australian Immunisation Register (AIR) or My Health Record (MHR)?

	Yes	No	Don't know
Australian Immunisation Register	◯	◯	◯
My Health Record	◯	◯	◯

Is your facility registered for My Health Record?
◯ Yes
◯ No
◯ Don't know

Can your facility software access My Health Record?
◯ Yes
◯ No
◯ Don't know

Do staff know how to look up residents' immunisation history using My Health Record?
◯ Yes
◯ No
◯ Don't know

What clinical software does your facility use?
____

Do you have staff registered to access the Australian Immunisation Register using PRODA?
◯ Yes
◯ No
◯ Don't know

Do these staff know how to look up residents' immunisation history in the Australian Immunisation Register?
◯ Yes
◯ No
◯ Don't know

Do these staff know how to upload vaccinations that have been given to residents into the Australian Immunisation Register?
◯ Yes
◯ No
◯ Don't know

Further comments about PRODA/AIR:
____

How do you find out a resident's vaccination history if they don't have a Medicare card?
____

Do your residents have their individual GPs or are there a few GPs who look after all the residents in your facility?
◯ Each resident has their own GP
◯ A few GPs look after all the residents in the facility
◯ Other

Can you please explain?
____

How many GPs look after all the residents in your facility?
____

Do the GPs visit the facility to routinely review the residents (i.e. not just when the residents are acutely unwell)?
◯ Yes
◯ No
◯ Other

Can you please explain?

____

Can you please provide a list of the GPs who review the residents in your facility?

____

Who normally administers vaccinations to your residents?
(Select all that apply)

- ☐ Resident's own GP
- ☐ Facility GP
- ☐ RACF nurse
- ☐ Authorised nurse immuniser (ANI) working at the RACF
- ☐ Pharmacist
- ☐ Other
- ☐ Don't know

Who else administers vaccinations to your residents?

____

Are vaccinations given in the facility or off-site?

- ◯ Mainly off-site
- ◯ Mainly in the facility

Can residents receive vaccinations in the facility?

- ◯ Yes
- ◯ No
- ◯ Don't know

What vaccinations are administered in the facility to your residents?

- ☐ COVID-19
- ☐ Influenza
- ☐ Pneumococcal
- ☐ Shingles

What is your facility's process for providing a vaccination on-site?
E.g. signed consent form prior to day of vaccination, designated room to administer vaccinations, vaccination clinics by contractor, system to monitor for side effects

____

Does your facility have a qualified Authorised Nurse Immuniser (ANI - registered nurse who can administer vaccines without a doctor present) or nurse practitioner?

- ◯ Yes - ANI
- ◯ Yes - nurse practitioner
- ◯ No
- ◯ Don't know

Does the ANI feel confident to administer vaccinations in the facility?

- ◯ Yes
- ◯ No
- ◯ Not applicable
- ◯ Don't know

Further comments about ANI? (optional)

____

Do you have difficulties accessing vaccination providers (e.g. GPs)?

- ◯ Yes
- ◯ No
- ◯ Don't know

Why is that?

____

Are you aware that participating pharmacies can administer vaccinations?

◯ Yes
◯ No

Do you know how to arrange vaccinations from these pharmacies?

◯ Yes
◯ No

Further comments (pharmacies):

____

Does your facility report to the Australian Immunisation Register when a resident has received a vaccination?

◯ Yes
◯ No - the vaccination provider does this
◯ Don't know

Are your staff confident to report to the Australian Immunisation Register?

◯ Yes
◯ No

Does your facility have a vaccine fridge on site?

◯ Yes
◯ No
◯ Don't know

Does the vaccine fridge have a data logger?

◯ Yes
◯ No
◯ Don't know

Are staff trained in appropriate cold chain management procedures?
E.g. cold chain requirements, vaccine fridge monitoring requirements, how to manage cold chain breaches

◯ Yes
◯ No
◯ Don't know

Have all relevant staff members completed the HETI Cold Chain Management online course?

◯ Yes
◯ No
◯ Don't know

Does your facility have a system to track when residents' vaccinations are due?

◯ Yes
◯ No
◯ Don't know

What is your system of tracking when vaccinations are due?

____

Are staff able to work out a catch-up immunisation schedule for a resident if needed?

◯ Yes
◯ No
◯ Don't know

Do you report your facility's resident vaccination rates to the Commonwealth government for these vaccinations?

	Yes	No	Don't know
COVID-19	◯	◯	◯
Influenza	◯	◯	◯
Pneumococcal	◯	◯	◯
Shingles	◯	◯	◯

How do you work out or calculate your residents' vaccination rate for these vaccinations?
eg. manually, run a report using software, for COVID-19 are rates determined by 6-monthly booster recommendations? etc. ______________________________

From the following list, please rank in order which issues are causing the most difficulties for your facility when vaccinating residents (from 1 to 5, 1=most most difficult, and 5=least difficult):

	1	2	3	4	5	Not applicable
Difficulty getting residents' immunisation history	◯	◯	◯	◯	◯	◯
No system to monitor when residents' vaccinations are due	◯	◯	◯	◯	◯	◯
Difficulty getting relatives to sign consent form	◯	◯	◯	◯	◯	◯
Resident or family refusing vaccination	◯	◯	◯	◯	◯	◯
Difficulty getting a vaccination provider	◯	◯	◯	◯	◯	◯

What do you think are the major issues stopping residents from getting vaccinated? ______________________________

What do you think helps residents get their vaccinations on time? ______________________________

Key points/issues identified during survey (PHU to complete): ______________________________

RACF resident vaccination project - Resident questionnaire

PROVIDE THE INFORMED CONSENT LETTER.

The Public Health Unit is interested to find out from people who live in aged care facilities what they think about vaccinations.

Your responses are valuable in helping us understand and address the barriers to vaccination in aged care facilities.

Your answers are confidential, and will not be shared with staff or other residents at your facility.

You do not have to answer any questions if you do not wish to.

You can stop at any time.

Do you have any questions before we start?

I consent to participating in this survey.
- ◯ Yes
- ◯ No

Name of the RACF: ______________________

Interview date: ______________________

PHU staff member completing the survey: ______________________

Resident's initials (optional) ______________________

How old are you?
- ◯ Under 65
- ◯ 65-69
- ◯ 70-74
- ◯ 75-79
- ◯ 80+
- ◯ Prefer not to say

What is your gender?
- ◯ Female
- ◯ Male
- ◯ Other

Do you identify as Aboriginal or Torres Strait Islander?
- ◯ Yes
- ◯ No
- ◯ Prefer not to say

What country were you born in? ______________________

What language(s) do you speak? (Select all that apply)	☐ English<br>☐ Other
What other languages do you speak?	__________________________
How long have you been in this facility?	○ Less than 6 months<br>○ 6 months to 1 year<br>○ 1-3 years<br>○ More than 3 years
Do you have any of these medical conditions? (Select all that apply)	☐ Heart disease<br>☐ Lung disease<br>☐ Diabetes<br>☐ Weak immune system<br>☐ Cancer<br>☐ None of the above<br>☐ Don't know

Are you aware of the following vaccine recommendations for older adults?
• COVID-19 vaccine every 6-12 months for adults aged 65-74 years, and every 6 months for adults aged 75 years and over
• Influenza (flu) vaccine every year for non-Indigenous adults from 65 years and any age for Indigenous adults
• Pneumococcal vaccine at 70 years for non-Indigenous adults, and 50 years for Indigenous adults
• Shingles vaccine at 65 years for non-Indigenous adults, and 50 years for Indigenous adults

	Yes	No
COVID-19	○	○
Influenza	○	○
Pneumococcal	○	○
Shingles	○	○

Where do you usually get information about vaccinations? (Select all that apply)	☐ GP, nurse or pharmacist<br>☐ Family or friends<br>☐ RACF staff<br>☐ News sources (e.g. TV, radio, newspapers)<br>☐ Religious or community leaders<br>☐ Internet<br>☐ Other<br>☐ Don't know<br>☐ Don't wish to answer
Where else do you get information about vaccinations?	__________________________
Have you ever received a vaccination in this facility?	○ Yes<br>○ No
Further comments (optional):	__________________________

How did facility staff obtain consent for your vaccination? __________________________________

Did staff give you enough information about vaccinations when getting your consent?
◯ Yes
◯ No

What other information would you have liked? __________________________________

If you get these infections (COVID-19, influenza, pneumococcal disease or shingles) as an older person, do you think you are likely to be more unwell (compared to a younger person)?
◯ Yes
◯ No
◯ Don't know
◯ Don't wish to answer

How effective do you think vaccines are in preventing you from falling sick from the infection?

	Very effective	Somewhat effective	Neutral	Somewhat ineffective	Very ineffective
Effectiveness of vaccine	◯	◯	◯	◯	◯

Further comments (optional): __________________________________

Do you think vaccines protect you from needing hospital admission or from serious illness if you catch the infection?
◯ Yes
◯ No
◯ Don't know
◯ Don't wish to answer

Further comments (optional): __________________________________

Have you ever had any bad experiences after previous vaccinations?
☐ No
☐ Pain/swelling/redness at the injection site
☐ Systemic symptoms (e.g. fever)
☐ Scared of needles
☐ Other
☐ Don't wish to answer

What was the reaction? __________________________________

Are you happy to consent to the facility and receive all four vaccinations recommended for your age?

	Yes	No	Don't know	Don't wish to answer
COVID-19	◯	◯	◯	◯
Influenza	◯	◯	◯	◯

Pneumococcal	○	○	○	○
Shingles	○	○	○	○

Further comments (optional): ______________________

If you are not willing to receive one or more of the four vaccinations, can you please explain why? (Select all that apply)

- ☐ Don't understand why I need the vaccination
- ☐ Too many vaccines
- ☐ Have to be vaccinated too often
- ☐ Don't trust vaccine companies
- ☐ I prefer natural immunity (from getting the infection)
- ☐ I worry about the possible side effects of vaccination
- ☐ I don't know where to get vaccinated
- ☐ Hard to go to the GP to get the vaccination
- ☐ It is too much trouble to get vaccinated
- ☐ Cultural or religious reasons
- ☐ Other
- ☐ Don't wish to answer

What are the other reasons? ______________________

What would make you more likely to get a vaccination? (Select all that apply)

- ☐ More information about the benefits and risks of vaccination
- ☐ Reassurance about the safety and effectiveness of vaccination
- ☐ Easier access to vaccination services (e.g. transport)
- ☐ Vaccination clinics in my facility
- ☐ Support from family or friends
- ☐ Recommendation from a healthcare provider
- ☐ Recommendation or support from my facility
- ☐ Knowing it helps protect my fellow residents
- ☐ Other residents getting the vaccination
- ☐ Other

What other reasons would make you more likely to get a vaccination? ______________________

Where have you gotten your vaccinations in the past? (Select all that apply)

- ☐ GP
- ☐ At my facility
- ☐ Pharmacy
- ☐ Other
- ☐ Don't know
- ☐ Don't wish to answer

Where else do you get your vaccinations? ______________________

How convenient is it for you to get your vaccination at these locations?

	Convenient	Not convenient	Don't know
GP	◯	◯	◯
Facility	◯	◯	◯
Pharmacy	◯	◯	◯

Did you know that some pharmacists can also give you vaccinations?

◯ Yes
◯ No
◯ Don't know
◯ Don't wish to answer

Would you be willing to have your vaccinations from your local pharmacist?

◯ Yes
◯ No
◯ Don't know
◯ Don't wish to answer

Why not?

____

What makes it hard for you to get a vaccine? (Select all that apply)

☐ I can't go on my own (I have a physical limitation)
☐ I don't know where to go to get vaccinated
☐ I'm not eligible or due to get a vaccine
☐ I have a medical reason for not getting vaccinated (e.g. allergy to vaccine)
☐ It is difficult to find or make an appointment
☐ Other

What other reasons make it hard for you to get vaccinated?

____

What would make it easier for you to get vaccinated? (Select all that apply)

☐ Transport
☐ Vaccination clinics in my facility
☐ Family support
☐ More information about the benefits and risks of vaccination
☐ Reassurance of safety and effectiveness of vaccines
☐ Recommendation from my GP
☐ Other
☐ Don't wish to answer

What other ways will make it easier for you to get your vaccinations?

____

Did you know that most of the vaccinations recommended for older adults are free?

◯ Yes
◯ No
◯ Don't wish to answer

Would you be willing to get vaccinations if you had to pay for them? (In general)

◯ Yes
◯ No
◯ Depends on the cost
◯ Don't know
◯ Don't wish to answer

Further comments on vaccine cost (optional):	__________________________________
Would you be more likely to get your vaccinations on time if someone reminded you?	◯ Yes<br>◯ No<br>◯ Don't know<br>◯ Don't wish to answer
Further comments (optional):	__________________________________
Would you be more willing to have vaccinations if your facility routinely recommended it?	◯ Yes<br>◯ No<br>◯ Don't know<br>◯ Don't wish to answer
Further comments (optional):	__________________________________
Do you have any other comments on what stops you or your fellow residents from getting vaccinated?	__________________________________
Do you have any other comments on what helps residents get vaccinated?	__________________________________

RACF resident vaccination project - Family questionnaire

PROVIDE THE INFORMED CONSENT LETTER.

The Public Health Unit is interested to find out from families of people who live in aged care facilities what they think about vaccinations.

Your responses are valuable in helping us understand and address the barriers to vaccination in aged care facilities.

Your answers are confidential, and will not be shared with staff or residents at the facility.

You do not have to answer any questions if you do not wish to.

You can stop the questionnaire at any time.

Do you have any questions before we start?

I consent to participating in this survey.
- ◯ Yes
- ◯ No

Name of the RACF: ____________________

Interview date: ____________________

PHU staff member completing the survey: ____________________

Interviewee's initials (optional): ____________________

What is your relationship to the resident?
- ◯ Spouse
- ◯ Daughter or son
- ◯ Other

What is your relationship? ____________________

How old is your family member?
- ◯ Under 65
- ◯ 65-69
- ◯ 70-74
- ◯ 75-79
- ◯ 80+
- ◯ Prefer not to say

What is your family member's gender?
- ◯ Female
- ◯ Male
- ◯ Other
- ◯ Prefer not to answer

Do they identify as Aboriginal or Torres Strait Islander?	◯ Yes<br>◯ No<br>◯ Prefer not to answer
What country were they born in?	__________________
What language(s) do they speak?<br>(Select all that apply)	☐ English<br>☐ Other
What other languages do they speak?	__________________
How long has your family member lived at this facility?	◯ Less than 6 months<br>◯ 6 months to 1 year<br>◯ 1-3 years<br>◯ More than 3 years
Does your family member have any of these medical conditions?<br>(Select all that apply)	☐ Heart disease<br>☐ Lung disease<br>☐ Diabetes<br>☐ Weak immune system<br>☐ Cancer<br>☐ Don't know<br>☐ Prefer not to answer<br>☐ None of the above
Further comments (optional):	__________________

Are you aware of the following vaccines recommended for older adults?

- **COVID-19 vaccine every 6-12 months for adults aged 65-74 years, and every 6 months for adults aged 75 years and over**
- **Influenza (flu) vaccine every year for non-Indigenous adults from 65 years and any age for Indigenous adults**
- **Pneumococcal vaccine at 70 years for non-Indigenous adults, and 50 years for Indigenous adults**
- **Shingles vaccine at 65 years for non-Indigenous adults, and 50 years for Indigenous adults**

	Yes	No	Don't know
COVID-19	◯	◯	◯
Influenza	◯	◯	◯
Pneumococcal	◯	◯	◯
Shingles	◯	◯	◯

Further comments (optional): __________________

Where do you usually get your information about vaccinations?
(Select all that apply)

- ☐ GP, nurse or pharmacist
- ☐ Family or friends
- ☐ RACF staff
- ☐ News sources (e.g. TV, radio, newspapers)
- ☐ Religious or community leaders
- ☐ Internet
- ☐ Other
- ☐ Don't know
- ☐ Don't wish to answer

Where else do you get information about vaccinations?

____

Has your family member ever received a vaccination in their facility?

- ◯ Yes
- ◯ No
- ◯ Don't know

Further comments (optional):

____

How did the facility staff obtain consent from you to vaccinate your family member?

____

Did staff give you enough information about vaccination when getting your consent?

- ◯ Yes
- ◯ No
- ◯ Don't know

What other information would you have liked?

____

If your relative caught these infections (COVID-19, influenza, pneumococcal disease or shingles) as an older person, do you think they are more likely to become very unwell (compared to a younger person)?

- ◯ Yes
- ◯ No
- ◯ Don't know
- ◯ Don't wish to answer

Further comments (optional):

____

How effective do you think vaccines are in preventing residents in RACFs from falling sick from the infection?

	Very effective	Somewhat effective	Neutral	Somewhat ineffective	Very ineffective
Effectiveness of vaccine	◯	◯	◯	◯	◯

Further comments (optional):

____

Do you think vaccines protect the resident from needing hospital admission or becoming seriously unwell if they catch the infection?

- ◯ Yes
- ◯ No
- ◯ Don't know
- ◯ Don't wish to answer

Further comments (optional):

____

Has your family member ever had any bad experiences after previous vaccinations?

- ☐ No
- ☐ Pain/swelling/redness at the injection site
- ☐ Systemic symptoms (e.g. fever)
- ☐ Scared of needles
- ☐ Other
- ☐ Don't know
- ☐ Don't wish to answer

What was the reaction?

____

Who decides if your family member is vaccinated?

- ◯ Me (family member)
- ◯ Joint decision between me and the resident
- ◯ Other

Who makes the decision?

____

How did you decide for your family to be vaccinated or not?

____

Did you discuss your decision with anyone else? (Select all that apply)

- ☐ Family member
- ☐ Healthcare worker
- ☐ Other

Who did you discuss your decision with?

____

How confident are you that you have all the information to decide if your family member should be vaccinated?

- ◯ Very confident
- ◯ Somewhat confident
- ◯ Not confident

Further comments (optional):

____

Would you provide consent to the facility for your family member to receive all four vaccinations recommended for their age?

	Yes	No	Don't know	Don't wish to answer
COVID-19	◯	◯	◯	◯
Influenza	◯	◯	◯	◯
Pneumococcal	◯	◯	◯	◯
Shingles	◯	◯	◯	◯

Further comments (optional):

____

If you are not willing to consent for your family member to receive one or more of the four vaccinations, can you please explain why?
(Select all that apply)

- ☐ Don't understand why they need the vaccine
- ☐ Too many vaccines
- ☐ Have to be vaccinated too often
- ☐ Don't trust vaccine companies
- ☐ I prefer natural immunity (from getting the infection)
- ☐ I worry about the possible side effects of vaccination
- ☐ I don't know where the resident can get vaccinated
- ☐ Hard to go to the GP to get the vaccination
- ☐ It is too much trouble to get vaccinated
- ☐ Cultural or religious reasons
- ☐ Other
- ☐ Don't wish to answer

What are the other reasons?

____

What would make you more likely to consent for your family member to be vaccinated?
(Select all that apply)

- ☐ More information about the benefits and risks of vaccination
- ☐ Reassurance about the safety and effectiveness of vaccination
- ☐ Easier access to vaccination services (e.g. transport)
- ☐ Vaccination clinics in the facility
- ☐ Support from family or friends
- ☐ Recommendation from a healthcare provider
- ☐ Recommendation or support from the facility
- ☐ Knowing it helps protect other residents in the facility
- ☐ Other residents getting the vaccination
- ☐ Other

What other reasons would make you more likely to consent for them to be vaccinated?

____

Where has your family member gotten their vaccinations?
(Select all that apply)

- ☐ GP
- ☐ At their facility
- ☐ Pharmacy
- ☐ Other
- ☐ Don't know
- ☐ Don't wish to answer

Where else do they get their vaccinations?

____

Is it convenient for your family member to get their vaccination at these locations?

	Convenient	Not convenient	Don't know
GP	○	○	○
Facility	○	○	○
Pharmacy	○	○	○

Did you know that some pharmacists can also give vaccinations?

- ○ Yes
- ○ No
- ○ Don't know
- ○ Don't wish to answer

Would you be happy for the local pharmacist to give vaccinations to your family member?	◯ Yes<br>◯ No<br>◯ Don't know<br>◯ Don't wish to answer
Why not?	__________________________________
Further pharmacy comments (optional):	__________________________________
What makes it hard for your family member to get a vaccine?<br>(Select all that apply)	☐ They can't go on their own (e.g. physical limitation, can't drive)<br>☐ They don't know where to get vaccinated<br>☐ They are not eligible or due to get a vaccine<br>☐ They have a medical reason for not getting vaccinated (e.g. allergy to vaccine)<br>☐ It is difficult to find or make an appointment<br>☐ Other
What other reasons make it hard for them to get vaccinated?	__________________________________
What would make it easier for your family member to get vaccinated?<br>(Select all that apply)	☐ Transport<br>☐ Vaccination clinics in the facility<br>☐ Family support<br>☐ More information about the benefits and risks of vaccination<br>☐ Reassurance of safety and effectiveness of vaccines<br>☐ Recommendation from the resident's GP<br>☐ Other<br>☐ Don't wish to answer
What other ways will make it easier for your family member to get vaccinated?	__________________________________
Did you know that most of the vaccinations recommended for older adults are free?	◯ Yes<br>◯ No<br>◯ Don't wish to answer
Would you be willing for the resident to be vaccinated if you had to pay for them? (In general, for any vaccine, if required)	◯ Yes<br>◯ No<br>◯ Depends on the cost<br>◯ Don't know<br>◯ Don't wish to answer
Would your family member be more likely to get their vaccinations on time if someone reminded you?	◯ Yes<br>◯ No<br>◯ Don't know<br>◯ Don't wish to answer
Would you be more willing to consent for your family member to be vaccinated if the facility routinely recommended it?	◯ Yes<br>◯ No<br>◯ Don't know<br>◯ Don't wish to answer

Do you have any other comments on what stops residents from being vaccinated?

____

Do you have any other comments on what helps residents become vaccinated?

____
